# Multimodal management of hormonal and oncological progression in PTHrP-secreting pancreatic neuroendocrine tumours

**DOI:** 10.1530/EO-25-0085

**Published:** 2026-02-13

**Authors:** Alexandra Zueva, Ee Wen Loh, Shamiso Masuka, Jonathan Wadsley, John Newell-Price, Alia Munir

**Affiliations:** ^1^ University of Sheffield, Sheffield, United Kingdom; ^2^ Sheffield Teaching Hospitals NHS Foundation Trust, Sheffield, United Kingdom; ^3^ Weston Park Cancer Centre, Sheffield, United Kingdom

**Keywords:** Pan-NET, humoural hypercalcaemia of malignancy, parathyroid hormone-related peptide, PTHrP-secreting tumour, neuroendocrine tumours

## Abstract

**Summary:**

Parathyroid hormone-related peptide (PTHrP)-secreting pancreatic neuroendocrine tumours (Pan-NETs) are a rare cause of humoural hypercalcaemia of malignancy (HCM). We report two contrasting cases of metastatic well-differentiated PTHrP-secreting Pan-NETs (WHO grade 2; Ki-67: 7 and 8%, respectively), highlighting the variability in disease progression, response to multiple treatment modalities, and long-term outcomes. The first patient, a 55-year-old woman with mild hypercalcaemia who was largely asymptomatic except for a persistent dry cough at presentation, achieved stable disease control following eight years of treatment with somatostatin analogues (SSAs), peptide receptor radionuclide therapy (PRRT), and chemotherapy. During a prolonged period of uncontrolled hypercalcaemia prior to chemotherapy, she developed rapid bilateral hip osteoarthrosis and tumour calcification, a rare complication from long-standing calcium elevation. The second patient, a 34-year-old woman, had a more aggressive disease course, requiring multiple hospital admissions for refractory moderate-to-severe hypercalcaemia and variceal bleeding. Despite initial tumour stabilisation following PRRT, she developed refractory hypercalcaemia, demonstrating only partial response to zoledronate, high-dose denosumab, and maximal somatostatin analogue therapy, and ultimately succumbed to progressive disease and metabolic deterioration. These cases underscore the heterogeneity of PTHrP-secreting Pan-NETs and the challenges in optimising treatment strategies, given the lack of data on the optimal sequencing of therapies. Notably, calcium can serve as a reliable tumour marker for disease control, with persistent severe hypercalcaemia being a potential prognostic factor of poor outcome in patients with PTHrP-related hypercalcaemia. International collaboration and knowledge exchange are needed to ascertain the most effective management of this rare functional syndrome.

**Learning points:**

- Consider PTHrP secretion in the context of hypercalcaemia in Pan-NETs.
- Calcium level can serve as a tumour marker in patients with PTHrP-related hypercalcaemia for assessing treatment response, disease progression, and tumour aggressiveness, with persistent hypercalcaemia being a prognostic factor for poor outcome.
- Hypercalcaemia may be resistant to standard treatments, and higher doses of denosumab may be necessary to achieve adequate control.
- An individualised multimodal treatment approach, including SSAs, PRRT, targeted therapy, chemotherapy, or a combination thereof, should be utilised to control PTHrP-secreting Pan-NETs.
- Although the optimal sequencing of these therapies remains unclear, multidisciplinary team discussions and shared decision-making are essential in managing such complex cases.

## Background

Parathyroid hormone-related peptide (PTHrP)-secreting pancreatic neuroendocrine tumours (Pan-NETs) are an exceptionally rare cause of humoural hypercalcaemia of malignancy (HCM). Unlike other functioning Pan-NETs, PTHrP secretion can cause life-threatening hypercalcaemia, requiring a multimodal approach for effective management. Patients often receive multiple treatment interventions to achieve tumour and hormonal control. Therapies include somatostatin analogues (SSAs), peptide receptor radionuclide therapy (PRRT), targeted therapy (everolimus, sunitinib, and cabozantinib), and chemotherapy. This is alongside standard calcium-lowering supportive measures, including hydration, bisphosphonates, denosumab, and calcitonin (1, 2). There is a paucity of data for the optimal treatment sequence for Pan-NETs and in particular for such rare functional syndromes. Here, we report two contrasting cases of metastatic PTHrP-secreting Pan-NETs presenting to an ENETS centre of excellence, highlighting the spectrum of disease presentation, management of hormonal and tumour progression, and treatment outcomes.

## Case 1

### Case presentation

A 55-year-old woman presented to the respiratory team with an 8-month history of persistent dry cough. She denied weight loss, abdominal pain, flushing, diarrhoea, or other systemic symptoms. The patient had no notable past medical or family history.

### Investigation

**Figure 1:**
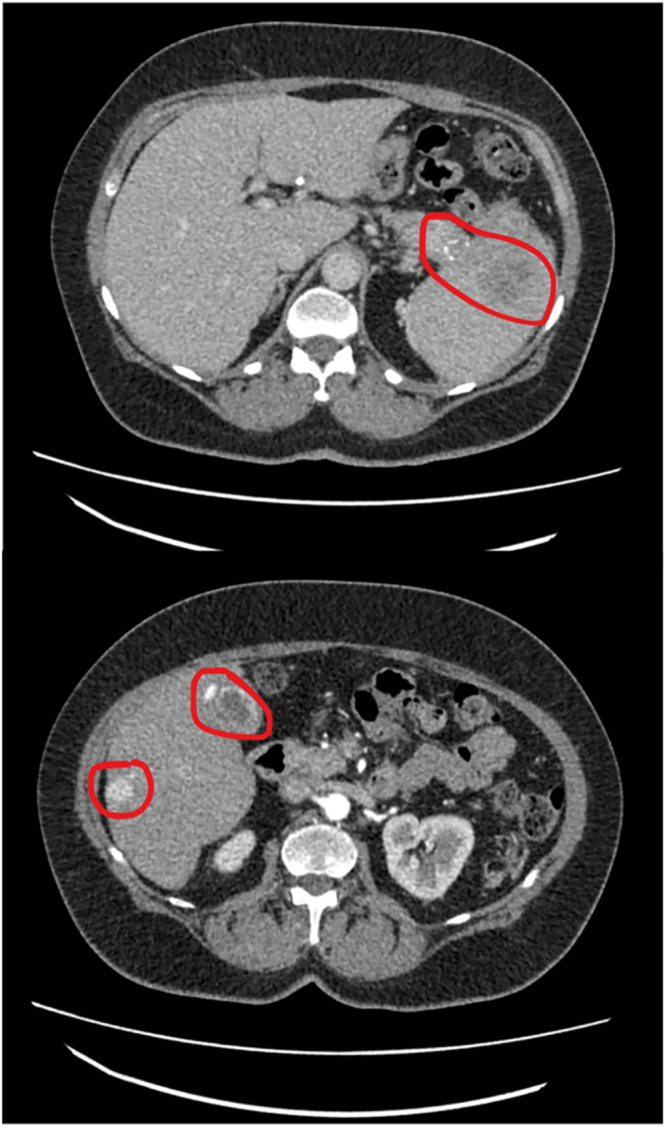
Case 1: contrast-enhanced axial CT images of the abdomen showing calcified pancreatic tail mass with extension into the spleen, along with multiple liver metastases.

Computed tomography pulmonary angiogram (CTPA) was performed to exclude pulmonary embolism and incidentally revealed a large pancreatic tumour. Further staging imaging revealed an 8 cm pancreatic mass, with invasion of the spleen, splenic vein occlusion, and multiple liver metastases. Notably, the lesions were calcified (Fig. 1). Laboratory tests showed mild hypercalcaemia (adjusted calcium 2.85 mmol/L, reference range: 2.14–2.56 mmol/L). Clinically, mild hypercalcaemia is defined as <3.0 mmol/L, moderate as 3.0–3.5 mmol/L, and severe as >3.5 mmol/L (1). PTH was suppressed (0.8 pmol/L, reference range: 1.6–6.9 pmol/L), and PTHrP was elevated (2.2 pmol/L, reference range: 0–1.8 pmol/L), with normal 25-hydroxyvitamin D levels (64.7 nmol/L). A full gut hormones profile is summarised in Table 1.

**Table 1:** Laboratory test results at presentation (case 1).

	Reference range	Result
PTHrP, pmol/L	0–1.8	**2.2**
Chromogranin A, pmol/L	0–59	**>300**
Gastrin, pmol/L	0–39	25
VIP, pmol/L	0–29	5
Glucagon, pmol/L	0–49	**61**
Pancreatic polypeptide, pmol/L	0–299	**>500**
CA 19-9, KU/L	0–35	16

Values in bold are above the reference range.

A liver biopsy confirmed a well-differentiated WHO grade 2 Pan-NET (Ki-67: 7%), with positive immunohistochemical staining for synaptophysin, chromogranin, and focal positivity for C19-9 and carcinoembryonic antigen (CEA). Nuclear medicine imaging using ^111^In-pentetreotide (Octreoscan®) demonstrated high somatostatin receptor expression (Krenning score 4). There was no evidence of bone metastases on imaging.

### Treatment

**Figure 2:**
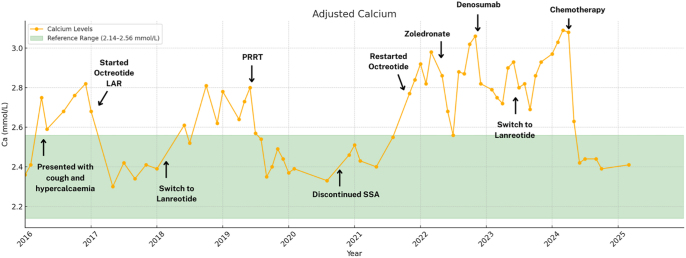
Case 1: the graph shows adjusted serum calcium level trends (yellow line) from 2016 to 2025. The green area indicates the reference range (2.14–2.56 mmol/L). Key events, such as medication initiation, are indicated in bold.

Initial treatment with octreotide long-acting repeatable (LAR, 30 mg intramuscularly monthly) resulted in an excellent response with normocalcaemia (Fig. 2). However, the patient experienced severe sleep disturbance as an unusual side effect. Switching to lanreotide (120 mg deep SC monthly) failed to improve her insomnia and did not effectively control hypercalcaemia. Disease progression, as evidenced by tumour growth, recurrent hypercalcaemia, and possibly tumour-related persistent cough, prompted PRRT with lutetium (^177^Lu) oxodotreotide, in line with recommendations from the multidisciplinary team (MDT). The patient underwent four cycles (7.4 GBq each), leading to tumour shrinkage and normalisation of calcium and PTH, lasting over two years. PTHrP assay was no longer available at that time. Despite stable disease, she experienced significant sleep disturbance, which adversely affected her quality of life. A trial discontinuation of SSA therapy led to marked improvement in her sleep, suggesting a treatment-related side effect.

Subsequent prolonged moderate hypercalcaemia was resistant to zoledronate and denosumab, leading to the rapid development of bilateral hip osteoarthrosis, an unusual complication, ultimately necessitating joint replacements.

With evidence of disease progression and persistent hypercalcaemia, SSAs were retried but remained ineffective. Consequently, the patient was initiated on alkylating-based chemotherapy with capecitabine and temozolomide (CapTem), following a MDT review.

The patient also developed type 3c diabetes mellitus, likely attributable to both tumour burden and SSA use, which was well managed with metformin and later insulin degludec. She was also commenced on pancreatic enzyme replacement therapy to address exocrine insufficiency.

In addition, follow-up imaging revealed gastric varices attributed to non-cirrhotic portal hypertension, for which she was commenced on non-selective beta-blockers.

### Outcome and follow-up

After two cycles of CapTem, imaging showed tumour reduction and stable normocalcaemia. However, she developed severe depression, which was managed with citalopram and required a treatment break. Currently, eight years after her initial diagnosis, the patient remains stable on monthly lanreotide injections.

## Case 2

### Case presentation

A 34-year-old woman presented with a two-month history of 19 kg weight loss with diarrhoea, nausea, and occasional vomiting. The patient had no significant past medical history. Her family history was notable for breast cancer in her mother, paternal aunt, and grandmother.

### Investigation

**Figure 3:**
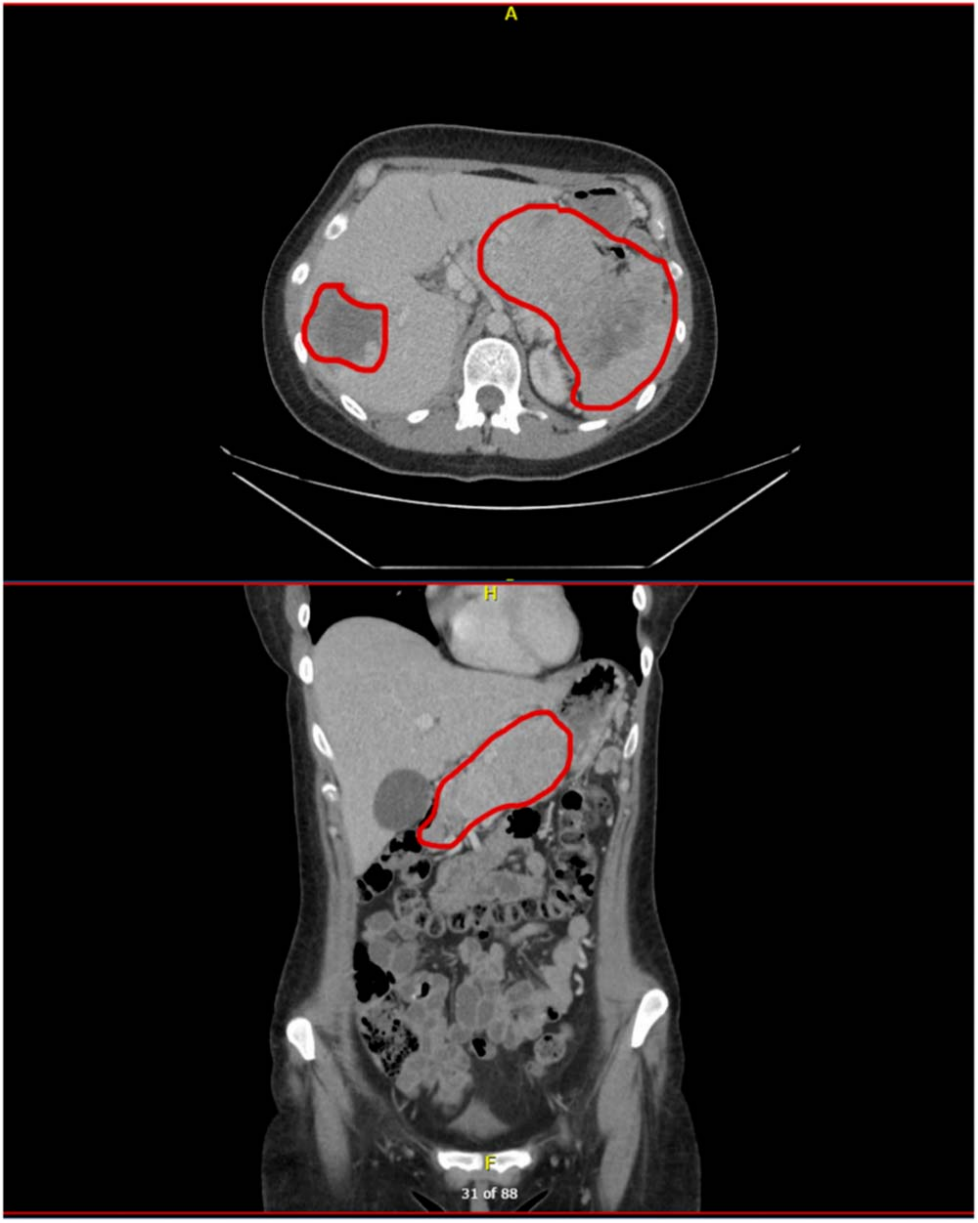
Case 2: contrast-enhanced axial (top) and coronal (bottom) CT images showing a large pancreatic mass, involving the body and tail, extending into the spleen and splenic flexure of the colon, with a large necrotic liver metastasis.

CT scan demonstrated a 13 cm inoperable pancreatic mass, invading the spleen, compressing the splenic vein and encasing the splenic artery, along with multiple oesophageal and gastric varices. There were also large necrotic liver metastases (Fig. 3).

Biochemical analysis showed moderate hypercalcaemia (adjusted calcium 3.09 mmol/L) with suppressed PTH (0.4 pmol/L) and normal 25-hydroxyvitamin D (52.1 nmol/L). PTHrP level was not measured due to the lack of available PTHrP assay at the time. The gut hormones profile revealed a mild elevation in vasoactive intestinal polypeptide (51 pmol/L, reference range: 0–29 pmol/L) and a substantial increase in somatostatin (716 pmol/L, reference range: 0–149 pmol/L) levels, consistent with the advanced Pan-NET (Table 2). No bone lesions were identified on imaging.

**Table 2:** Laboratory test results at presentation (case 2).

	Reference range	Result
Chromogranin A, pmol/L	0–59	**192**
Chromogranin B, pmol/L	0–149	**216**
Gastrin, pmol/L	0–39	3
VIP, pmol/L	0–29	**51**
Glucagon, pmol/L	0–49	31
Somatostatin, pmol/L	0–149	**716**
Pancreatic polypeptide, pmol/L	0–299	15
5-HIAA (blood), nmol/L	0–139	46

Values in bold are above the reference range.

**Figure 4:**
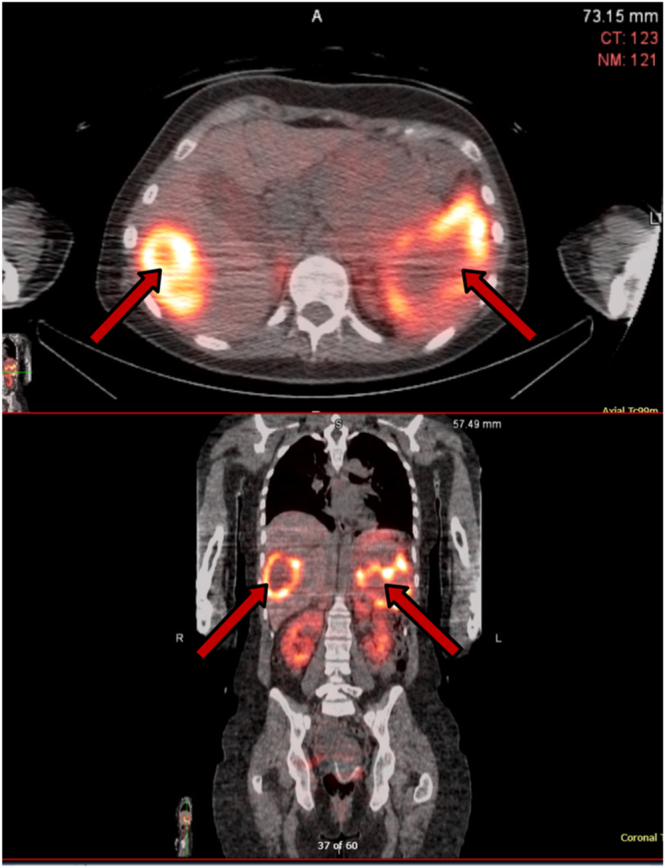
Case 2: octreotide scan with SPECT/CT images in axial (top) and coronal (bottom) views showing intense radiotracer uptake in a pancreatic mass and a liver metastasis (red arrows), consistent with Krenning 4 avidity.

A primary mass biopsy verified a well-differentiated WHO grade 2 Pan-NET (Ki-67: 8%), positive for synaptophysin, chromogranin, and AE1/AE3. Octreoscan® showed positive uptake with Krenning score 4 (Fig. 4).

### Treatment

The patient was commenced on monthly lanreotide 120 mg deep SC injections. Due to disease progression, she received four cycles of lutetium (^177^Lu) oxodotreotide.

**Figure 5:**
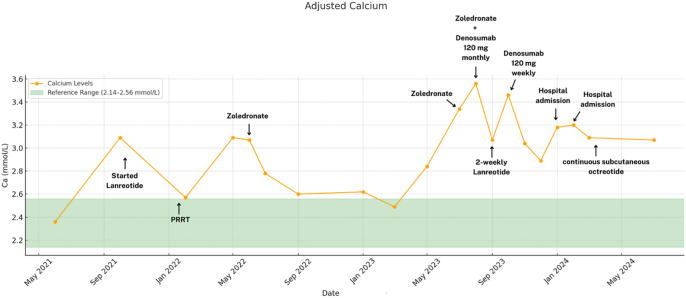
Case 2: the graph shows adjusted serum calcium level trends (yellow line) from June 2021 to July 2024. The green area indicates the reference range (2.14–2.56 mmol/L). Key events, such as medication initiation and hospital admissions, are marked in bold. Zoledronate was administered at 4 mg IV.

Following PRRT, her disease stabilised, with mild shrinkage of liver metastases and reduced uptake on SPECT-CT. However, she experienced recurrent symptomatic hypercalcaemia, presenting with lethargy, confusion, polyuria, and polydipsia, necessitating multiple hospital admissions (Fig. 5). Despite monthly infusions of intravenous zoledronate, she experienced refractory severe hypercalcaemia, prompting a switch to monthly subcutaneous denosumab 120 mg injections. As hypercalcaemia remained poorly controlled, treatment was intensified to two-weekly and eventually once-weekly denosumab (120 mg), along with two-weekly lanreotide (120 mg) injections, allowing temporary control of calcium levels.

Development of portal hypertension led to multiple episodes of profound upper gastrointestinal bleeding secondary to oesophageal and gastric varices. Endoscopic interventions with sclerotherapy and blood transfusions were performed.

The patient’s disease was further complicated by the development of type 3c diabetes mellitus, presenting mainly with lethargy and hyperglycaemia (28 mmol/L) in the absence of ketonaemia. Diabetes was successfully managed with basal-bolus insulin therapy (detemir and lispro).

Sunitinib and other active treatments, including chemotherapy, were deemed unsuitable due to the risk of variceal bleeding and the patient’s declining performance status. Given the complexity of the case, all treatment decisions were guided by MDT discussions.

### Outcome and follow-up

In view of rapid clinical deterioration and refractory hypercalcaemia, the patient was transitioned to palliative care. During the last four months of life, she received continuous subcutaneous octreotide (1.5 mg/24 h) via a syringe driver and daily home fluid administration, along with weekly denosumab to manage hypercalcaemia and prevent further hospitalisations. She died three years after her original presentation, at the age of 37.

## Discussion

Despite both patients having metastatic PTHrP-secreting Pan-NETs with apparently similar tumour histology, their disease progression varied significantly. This heterogeneity underscores the need for an individualised multidisciplinary treatment approach and highlights the importance of recognising specific tumour features that may predict clinical aggressiveness.

Interestingly, the first patient remained largely asymptomatic, except for a persistent cough, which preceded disease progression and subsided upon disease stabilisation. The association between PTHrP secretion and cough remains unclear. However, a tumour co-secretion of vasoactive peptides, such as bradykinin, is a possible explanation. While bradykinin is known to enhance cough reflex sensitivity, this mechanism remains speculative in Pan-NETs as no direct biochemical confirmation has been obtained and, to our knowledge, it has not been described in the literature.

These cases demonstrate that calcium levels can serve as a reliable tumour marker to assess disease progression and treatment response, particularly when PTHrP assays are unavailable. Notably, persistent hypercalcaemia despite multimodal therapy appears to be a prognostic factor for aggressive disease and poorer outcome.

Elevated systemic calcium levels may contribute to tumour calcification, as observed in case 1. Such a radiological finding is uncommon but has been recognised in pancreatic tumours (3). Further research is needed to determine whether calcification could serve as a diagnostic or prognostic marker in NETs.

It remains poorly understood whether the patient’s osteoarthrosis could be driven by hypercalcaemia or direct PTHrP action. While physiologic PTHrP regulates chondrocyte differentiation and proliferation, persistently elevated PTHrP levels secreted by tumours may disrupt normal cartilage and bone metabolism (4).

In patients with Pan-NETs presenting with hypercalcaemia, it is essential to rule out primary hyperparathyroidism (PHPT), particularly in the context of MEN-1 (2). Suppressed PTH in both cases effectively excluded PHPT, which is typically characterised by elevated or inappropriately normal PTH.

Younger age at presentation may contribute to a more aggressive disease course, due to possible underlying genetic predispositions, although no genetic testing was performed in the current case due to the absence of syndromic features or a family history of endocrine tumours.

Other important differentials include granulomatous diseases, such as sarcoidosis, and ectopic 1,25-dihydroxyvitamin D (calcitriol) production, most commonly associated with lymphomas (1). Unfortunately, calcitriol levels were not assessed in our cases. In addition, the lack of biochemical confirmation of PTHrP secretion in case 2, due to assay unavailability, represents a limitation. However, PTHrP-related hypercalcaemia was strongly suspected based on suppressed PTH, the absence of bone metastases, and the relative likelihood of this mechanism compared to calcitriol-mediated hypercalcaemia.

A single-site biopsy may not reliably represent the overall tumour grade, which can vary between primary tumour and metastatic lesions or increase over time. Bourdeleau *et al.* (5) demonstrated temporal intra-tumour heterogeneity in Ki-67 index and found that an increase of 2% and more per year was associated with a twofold increased risk of death. Therefore, tumour grade in case 2 might have been higher than reported, aligning with its clinically aggressive behaviour.

In view of possible dedifferentiation over time and discordance between clinical features, imaging findings, and tumour grade, FDG PET/CT may be useful for assessing disease metabolic activity. A targeted biopsy from areas with higher glycolytic activity could potentially aid in risk stratification and prognostication.

The presented cases highlight the effectiveness of PRRT in controlling disease progression and achieving tumour reduction. Notably, preliminary results from NETTER-2, a phase 3 randomised trial, have demonstrated a significant improvement in progression-free survival (PFS) with lutetium (^177^Lu) oxodotreotide plus octreotide LAR compared to high-dose octreotide alone in newly diagnosed advanced higher grade 2–3 gastroenteropancreatic NETs, suggesting a potential role for PRRT as first-line therapy (6).

Given these findings, there is a potential therapeutic benefit in earlier PRRT initiation in functional Pan-NETs, rather than delaying the treatment until disease progression. In case 1, PRRT resulted in 26 months of stable disease with normocalcaemia. According to available data on similar cases, among 11 patients with PTHrP-secreting Pan-NETs and hypercalcaemia treated with PRRT, five achieved a biochemical response, with some also demonstrating tumour reduction (7).

The optimal treatment sequence remains under investigation. Both PRRT and CapTem chemotherapy are currently the mainstay treatment for advanced metastatic Pan-NETs (2). A recent retrospective study demonstrated comparable survival outcomes when either PRRT or CapTem was used as a second-line therapy, indicating the flexibility in treatment sequencing based on the patient’s characteristics and needs (8). However, prospective confirmation is needed.

CapTem showed efficacy in prolonging PFS and inducing tumour reduction, along with a favourable safety profile relative to streptozotocin-based chemotherapy (9). Similar case reports of PTHrP- and calcitriol-secreting NETs have demonstrated successful metastatic disease control with hypercalcaemia response and a marked reduction in PTHrP levels following CapTem, although the duration of the response is unclear (7).

Notably, chemotherapy with alkylating agents correlated with tumour grade progression and Ki-67 increase (5). Given this association, sequencing of treatments and the use of temozolomide, in particular, requires careful weighted consideration, balancing the potential short-term benefits with long-term consequences on tumour behaviour. It may be best to reserve chemotherapy as a last-resort option when other treatment modalities fail or are contraindicated, or when rapid tumour shrinkage is required.

Denosumab, a potent osteoclast inhibitor, is widely used in osteoporosis and HCM, particularly in cases refractory to IV bisphosphonates (1). In oncology, the standard dosing interval is four weeks. Given the risk of rebound bone loss and fractures, and rebound HCM upon denosumab discontinuation, the treatment, if tolerated, is typically continued indefinitely in these settings. One study showed the efficacy of a more intensive regimen of 120 mg weekly for four weeks, followed by monthly injections in patients with bisphosphonate-resistant HCM, with most patients responding to treatment (10). Importantly, our case describes the prolonged use of high-dose weekly denosumab for approximately 10 months without significant side effects, suggesting its relative safety in refractory humoural HCM. Further research is needed to determine the optimal sequencing of calcium-lowering treatments, especially in life-threatening cases resistant to standard therapy. There are no prospective head-to-head comparisons between zoledronate and denosumab in HCM treatment (1). However, denosumab has shown superiority in HCM prevention based on low-quality evidence and is preferred in patients with renal insufficiency. It is unknown whether denosumab may provide bone protection or prevention of HCM in PTHrPoma, even in cases of stable disease with normocalcaemia.

Although extremely rare, PTHrP-secreting NETs pose significant clinical challenges, particularly in managing refractory hypercalcaemia. However, individualised multimodal treatment strategies can improve long-term outcomes and prolong survival. Multidisciplinary care, especially with the advice from a centre of excellence, is crucial for comprehensive personalised care, improving outcomes and quality of life in these challenging cases.

Given the rarity of these secretory Pan-NETs, a collaborative effort to integrate and review world cases could enhance our understanding of these tumours and formulate most effective management strategies.

## Declaration of interest

The authors declare no conflict of interest that could be perceived as prejudicing the impartiality of this work.

## Funding

This work did not receive any specific grant from any funding agency in the public, commercial, or not-for-profit sector.

## Patient consent

Written informed consent for the publication of their clinical details and clinical images was obtained from the patient or relative of the patient where deceased.

## Author contribution statement

AZ collected clinical data, performed the literature review, and drafted the manuscript. EWL was involved in patient care and reviewed and edited the manuscript. SM was involved in patient care and manuscript review. JW was involved in patient care, contributed to clinical interpretation, and manuscript review and editing. JNP was involved in patient care as the consultant responsible for patient 2, contributed to interpretation, and reviewed and edited the manuscript. AM was involved in patient care as the consultant responsible for patient 1, supervised the project, contributed to interpretation, and reviewed and edited the manuscript. All authors have reviewed and approved the final version of the manuscript. For the reported cases, all authors either directly participated in patient care or obtained appropriate permission from the responsible clinicians involved in the management of the patients.
